# Real-World Analysis of Short-Term Effectiveness of Oral Semaglutide: Impact on Glycometabolic Control and Cardiovascular Risk

**DOI:** 10.3390/ph18060856

**Published:** 2025-06-08

**Authors:** Sara Palazzi, Federica Sentinelli, Antonella Zugaro, Sara Morgante, Livia Santarelli, Sandra Melanzi, Annamaria De Mutiis, Deamaria Piersanti, Barbara Macerola, Marco Iezzi, Pietro Mercuri, Alessandro Ferranti, Daniele Tienforti, Maria Gisella Cavallo, Arcangelo Barbonetti, Marco Giorgio Baroni

**Affiliations:** 1 Department of Clinical Medicine, Public Health, Life and Environmental Sciences (MeSVA), University of L’Aquila, 67100 L’Aquila, Italy; sara.palazzi@graduate.univaq.it (S.P.); federica.sentinelli@univaq.it (F.S.); alessandro.ferranti@graduate.univaq.it (A.F.); daniele.tienforti@graduate.univaq.it (D.T.); arcangelo.barbonetti@univaq.it (A.B.); 2 UOSD Diabetology, San Salvatore Hospital, ASL1 Abruzzo, 67100 L’Aquila, Italy; anzugaro@asl1abruzzo.it (A.Z.); morgante.sn@gmail.com (S.M.); 3 UOSD Diabetology, PO Castel di Sangro-Sulmona, ASL1 Abruzzo, 67031 L’Aquila, Italy; liviasantarelli@yahoo.it (L.S.); sandra.melanzi@asl1abruzzo.it (S.M.); annamaria.demutiis@gmail.com (A.D.M.); 4 UOSD Diabetology, POAvezzano, ASL1 Abruzzo, 67051 L’Aquila, Italy; dpiersanti@asl1abruzzo.it (D.P.); b.macerola@asl1abruzzo.it (B.M.); marco.iezzi@asl1abruzzo.it (M.I.); p.mercuri@asl1abruzzo.it (P.M.); 5 Department of Experimental Medicine, Sapienza University, 00185 Rome, Italy; gisella.cavallo@uniroma1.it; 6 Neuroendocrinology and Metabolic Diseases, IRCCS Neuromed, 86077 Pozzilli, Italy

**Keywords:** GLP1-receptor agonist, type 2 diabetes, HbA1c, BMI, weight, duration of diabetes

## Abstract

**Background**: Oral semaglutide, a GLP1-receptor agonist (GLP1-RA), shows promise in efficacy and compliance, especially amid the global shortage of injectable GLP-1 RAs. Its short-term effectiveness remains unexplored. **Objective**: This real-world observational study assessed the short-term effectiveness of oral semaglutide after three months of therapy. **Methods**: Patients with type 2 diabetes from four Italian diabetes centers, who received an initial prescription of oral semaglutide, were reassessed after three months. Primary outcomes included glycated hemoglobin (HbA1c) and body weight reduction; secondary outcomes involved changes in lipid parameters and cardiovascular risk. **Results**: Among 167 participants (mean age 66.5 years, mostly obese, baseline HbA1c 8.4% ± 1.5), 83.2% received a 7 mg dose. After three months, HbA1c significantly declined (8.4% to 7.1%, −1.3%, *p* < 0.001), alongside body mass index (BMI) (30.9 kg/m^2^ to 29.6 kg/m^2^, *p* < 0.0001). The target HbA1c ≤ 7% was achieved by 54.5%, and 34.7% reached ≤6.5%. Patients losing >5% of their initial weight (30.5%) saw the largest HbA1c drop (−1.9%). Those with newly diagnosed diabetes or a duration < 5 years showed superior responses (*p* = 0.001), while no significant differences were found based on the timing of drug administration. Oral semaglutide replaced or supplemented prior therapies, allowing discontinuation of dipeptidyl peptidase 4 inhibitors (DPP4i), sulfonylureas, glinides, and acarbose, and deprescription of thiazolidinediones. A significant reduction in cardiovascular risk was observed (*p* = 0.04), together with a significant reduction in lipid parameters. **Conclusions**: Oral semaglutide showed significant short-term efficacy, reducing HbA1c, body weight, and cardiovascular risk in three months, making it a valuable therapeutic option.

## 1. Introduction

Type 2 diabetes mellitus (T2DM) is a chronic condition associated with numerous cardiovascular risk factors (hypertension, obesity, and dyslipidemia) and the development of micro- and macrovascular complications, posing a significant public health issue. It is crucial to initiate prompt and aggressive treatment of diabetes early in the course of the disease, as recommended by guidelines, because delaying the intensification of anti-hyperglycemic therapy by just one year exposes patients to a 62% increased risk of cardiovascular disease overall and a 67% increased risk of myocardial infarction in the subsequent 5 years [1]. In the treatment of T2DM, a pro-active and advanced therapeutic strategy is necessary, utilizing drugs that effectively target hyperglycemia while positively affecting other cardiovascular (CV) risk factors. GLP-1 receptor agonists (GLP1-RA) play a crucial role in glycemic control and β-cell dysfunction, showing favorable effects on body weight, lipid profile, and blood pressure, with a low risk of hypoglycemia [2].

The majority of GLP1-RA are in an injectable form, but recently, the first and only oral GLP1-RA, semaglutide, has become available. The co-formulation of semaglutide with SNAC (Sodium N-(8-[2-hydroxybenzoyl] Amino) Caprylate) allows this peptide to overcome the obstacles determined by its intrinsic properties (hydrophilicity, low permeability, high molecular weight, enzymatic lability), making it absorbable through the gastric wall [3]. The PIONEER clinical trials program, encompassing 10 randomized controlled trials (RCTs) involving almost 10.000 individuals with type 2 diabetes (T2D), has consistently demonstrated the efficacy of oral semaglutide in improving glycemic control, reducing body weight (BW), controlling CV risk factors such as blood pressure, lipid profile, and inflammation markers, and ensuring drug safety [4,5,6,7,8,9,10,11,12,13]. These trials enrolled a heterogeneous population of patients with type 2 diabetes mellitus, ranging in age from approximately 55 to 70 years, with either short or long disease duration, who were overweight or obese and exhibited inadequate glycemic control (HbA1c between 8% and 8.3%). The findings demonstrated that oral semaglutide led to a significant reduction in HbA1c (from −1.2% in PIONEER 4 to −1.3% in PIONEER 2 and 3), accompanied by clinically meaningful weight loss (ranging from −2.6 kg in PIONEER 7 to −4.4 kg in PIONEER 4). These improvements occurred without an increased risk of hypoglycemia and were associated with a favorable tolerability profile, as evidenced by the low incidence of gastrointestinal adverse events such as nausea and vomiting. Concerning cardiovascular protection, the SOUL trial [14] represents a pivotal advancement in the clinical evaluation of oral semaglutide, focusing specifically on its cardiovascular safety and efficacy in patients with type 2 diabetes who are at high cardiovascular risk. The study found a 14% reduction in the risk of major adverse cardiovascular events (MACE) with oral semaglutide compared to placebo (hazard ratio, 0.86; 95% CI, 0.77–0.96). This benefit was consistent regardless of baseline use of SGLT2 inhibitors, indicating that oral semaglutide provides cardiovascular protection independent of concurrent therapies.

Although RCTs have high scientific relevance, they often face challenges in translating their results to real-world clinical settings. Since they are conducted under highly controlled conditions, the representativeness of patients included in the studies may be limited by the exclusion of elderly patients or those with complex medical conditions. Additionally, an improvement in the clinical condition can often be observed even in patients treated with a placebo, known as the “trial effect”. Therefore, it is crucial to consider evidence of efficacy and safety in everyday clinical practice, going beyond the ideal inclusion criteria of RCTs. From this perspective, in the clinical field and among regulatory agencies, observational data from everyday clinical practice, processed as real-world data (RWD) and real-world evidence (RWE), is gaining increasing importance. RWD observational analyses assess the efficacy and safety of drugs in larger and less-selected populations compared to RCTs. RWE observation further documents whether the drug is prescribed appropriately and used following guidelines, and identifies subgroups of patients who may benefit the most. Several RWD observations of oral semaglutide have been performed [15,16,17,18,19], all reporting safety and efficacy, which have also been shown in patients with a shorter disease duration, better glycometabolic control, and a higher average age. All studies have assessed efficacy and safety in the medium (6–12 months) or long term, but data on how long it takes for oral semaglutide to become effective are lacking. This is crucial for the early modification of cardiovascular risk factors associated with diabetes, thereby improving the natural course of the disease.

The aim of this study was, therefore, to evaluate, in a real-world dataset, the effectiveness of oral semaglutide over a short period of three months in patients with type 2 diabetes.

## 2. Results

### 2.1. Study Population

We identified 231 eligible patients at baseline, 53 of whom did not continue the study within the three-month established timeline and were withdrawn. A total of 178 patients attended the follow-up diabetes visits at three months, and among these, 11 patients (6.1%) experienced side effects that required discontinuation of the medication. These were primarily moderate-intensity gastrointestinal effects, including nausea and vomiting.

A total of 167 continued the medication for the entire study duration, allowing for the evaluation of changes in the clinical and biochemical parameters between the start of recruitment and three months later. Some biochemical parameters were not measured in all patients or at both time points. Therefore, only parameters available for each patient at baseline (T0) and after three months (T1) were considered.

The clinical characteristics and biochemical parameters at T0 and T1 are presented in Table 1.

**Figure 1:**
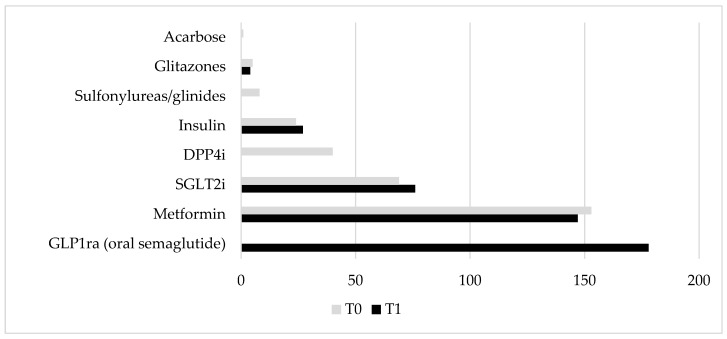
Graphical representation of changes in frequency of use of oral and/or injectable hypoglycemic agents before (T0, grey columns) and after (T1, black columns) introduction of oral semaglutide. Abbreviations: DPP4i: dipeptidyl peptidase 4 inhibitors, SGLT2i: sodium-glucose cotransporter 2 inhibitors, GLP1ra: glucagon-like peptide-1 receptor agonists.

The 167 participants, with an average age of 66.5 years, were predominantly male (54%) and had an average duration of diabetes of 7.6 ± 4.2 years. They were mainly obese individuals (average body mass index of 30.9 kg/m^2^) who started oral semaglutide with inadequate glycemic control (mean HbA1c of 8.4%, mean fasting plasma glucose of 173 mg/dL). At T0, the patients were undergoing various oral and/or injectable hypoglycemic therapies, and at the time of recruitment into the study, oral semaglutide was introduced either as an addition (65%) or as a replacement for their previous therapy (35%). In Figure 1, the drug classes are shown before and after the introduction of oral semaglutide. Specifically, there was an expected discontinuation of dipeptidyl peptidase 4 inhibitors (DPP4 inhibitors), a deprescribing of thiazolidinediones, and suspension of sulfonylureas, glinides, and acarbose. When sulfonylureas or glinides were discontinued, oral semaglutide was supplemented with either basal insulin (in 3 patients) or SGLT2 inhibitors (in 7 patients).

Oral semaglutide was started at a dosage of 3 mg for the first 4 weeks and then, based on each patient’s phenotype, increased to 7 mg (83.2%), maintained at 3 mg (12.6%), or gradually increased up to 14 mg (4.2%). The medication, taken after at least a few hours of fasting, was administered before breakfast in 17.4% of cases, before lunch in 35.9% of cases, and before dinner in 46.7% of cases.

### 2.2. Changes in Clinical and Biochemical Parameters at Follow-Up

After a 3-month follow-up, a Wilcoxon signed-rank test was applied to detect possible changes in HbA1c and BMI variables. We observed a significant reduction in HbA1c (−1.3%, from 8.4% to 7.1%, *p* < 0.000) and a significant change in BMI (from an obese phenotype, BMI 30.9 kg/m^2^, to an overweight phenotype, BMI 29.6 kg/m^2^, *p* < 0.000), as described in Table 1. Over half of the patients (54.5%) achieved an HbA1c of 7% or lower, and 58 out of 167 patients (34.7%) achieved an HbA1c of 6.5% or lower.

At baseline there were 28 patients with HbA1c ≤ 7%, 55 patients with HbA1c between 7 and 8%, 41 patients with HbA1c between 8 and 9%, and 43 patients with HbA1c > 9%. Those who started with the worst metabolic compensation (HbA1c > 9%) obtained the greatest delta of HbA1c reduction at T1 (mean delta reduction 3.05%) compared with other groups who obtained overall HbA1c reduction values of less than 1.2%.

Obese patients at baseline (a total of 80 subjects) achieved the greatest loss in body weight (−4.5 kg on average), while overweight or normal-weight subjects lost less than 2.3 kg body weight. Patients who lost more than 5% of their initial body weight (51/167, 30.5%) achieved the greatest reduction in HbA1c (−1.9%), while those who remained stable or gained weight (45/167, 26.9%) still achieved a good reduction in HbA1c, albeit to a lesser extent (−0.7%).

Based on the availability of other biochemical data collected, after a Wilcoxon signed-rank test, significant reductions were also observed in total cholesterol, low-density lipoprotein (LDL cholesterol), GOT, GPT, and microalbuminuria (Table 1).

### 2.3. Subgroup Analyses

**Figure 2:**
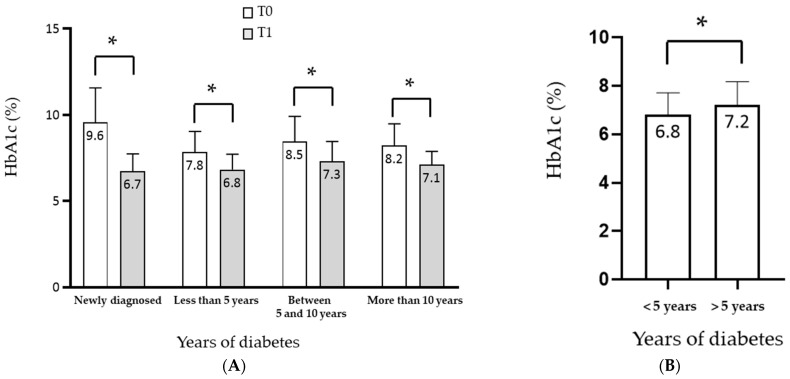
(**A**) Mean HbA1c values (expressed as percentages) at T0 (white columns) and T1 (grey columns) based on different classes of diabetes duration (* *p* < 0.001; paired *t*-test); (**B**) mean HbA1c values at T1 in subjects with diabetes diagnosis <5 years and >5 years (* *p* < 0.001; *t*-test).

With regard to the different groups created based on the duration of diabetes (newly diagnosed, <5 years, 5–10 years, >10 years), all groups showed a significant reduction in HbA1c at T1 (*p*-value 0.001) (Figure 2A). Furthermore, a particularly significant HbA1c reduction (−1.61%, *p* < 0.001) was observed at T1 in those with a diabetes duration of less than 5 years. Also, the Hba1c reduction at T1 was significantly greater in subjects with a duration < 5 years compared to subjects with a duration of > 5 years (6.8% and 7.2%, respectively, *p* < 0.007) (Figure 2B).

A significantly greater reduction in HbA1c was observed in the group of patients with a body weight loss of at least 5% compared to those who had a smaller weight loss (respectively, −1.9% vs. −1.06%).

No significant differences were found between the different timings of drug administration, although it was shown that lower metabolic control was achieved in the “before lunch” group (Table 2).

**Figure 3:**
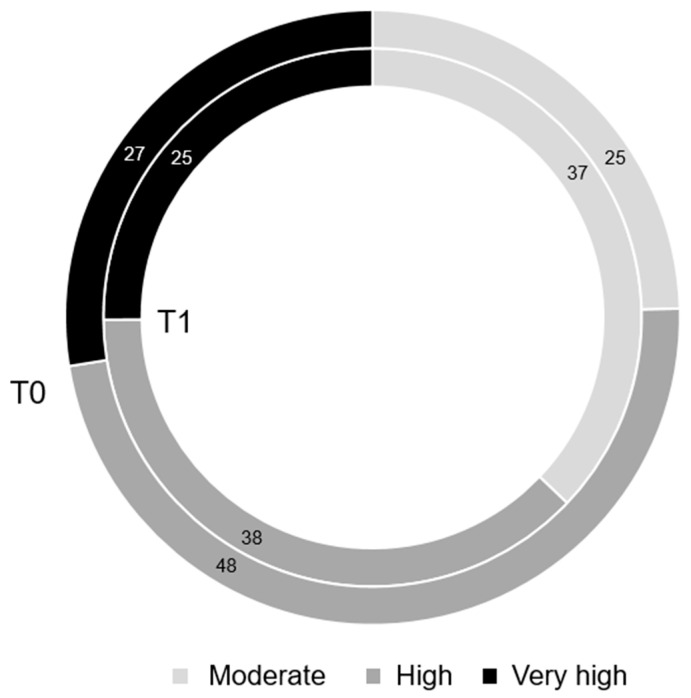
Changes in cardiovascular risk classes between T0 and T1. Data are reported as percentages.

With regard to the CV risk categories, a good percentage of patients at “high” (21.3%) or “very high” (8.7%) cardiovascular risk moved to a lower risk class, namely “moderate” and “high,” respectively (*p* value 0.04), as shown in Figure 3.

### 2.4. Multiple Linear Regression Analysis

Finally, to evaluate which variable was independently associated with HbA1c, we used multiple linear regression analysis to test the following variables: duration of diabetes, age, gender, BMI, triglycerides, and creatinine.

A significant independent association with HbA1c reduction was shown for shorter diabetes durations (*p*-value 0.028), independently from the other variables in the model; emphasizing the clinical significance of this observation is the fact that the medication works effectively at any age and initial BMI, but is more effective when started early in the disease (Table 3).

## 3. Discussion

This is the first real-world observational study to show the effectiveness of oral semaglutide after just 3 months of therapy. Oral semaglutide was prescribed to T2D patients, predominantly obese, with an initial mean HbA1c of 8.4%. HbA1c is recognized as the gold standard for evaluating diabetes control, and its reduction is a sign of improved glucose management [20]. After 3 months, a significant reduction in HbA1c (−1.3% or −14 mmol/mol) and body weight (−3.4 kg) was recorded, with 54.5% of patients achieving an HbA1c target of <7% and 30.5% of patients losing more than 5% of their initial body weight. Furthermore, those who lost at least 5% of their initial body weight achieved the greatest reduction in HbA1c, which in our study was nearly 2%, reinforcing the importance of weight loss in reaching the recommended HbA1c targets for type 2 diabetes. As shown in the PIONEER 3 study, great outcomes in terms of body weight and HbA1c reduction are maintained even at 78 weeks [6]. The fact that a diagnosis of diabetes < 5 years prior to the study was the strongest predictor of HbA1c reduction further highlights the need for an early proactive intervention, when beta-cell function and insulin resistance are still adjustable. In clinical situations requiring effective and rapid glycemic control, oral semaglutide can represent an excellent alternative to injectable drugs such as insulin or other GLP-1 RAs.

These excellent results were achieved in the majority of cases with the intermediate dosage of 7 mg, as only 4% of patients reached 14 mg, while 13% remained at 3 mg. Compared to other longer-duration studies [15,16,17,18,19], where a good percentage of patients reached 14 mg, in our shorter study, most patients remained at the intermediate dosage of 7 mg. As demonstrated in the IGNITE study [18], where one-third of patients were on 3 mg of oral semaglutide, after six months of follow-up an HbA1c reduction of 0.9% was achieved, highlighting the fact that even the lowest dosage of the drug can be effective. No significant differences emerged between drug intake before breakfast, before lunch, or before dinner. The group taking the medication before lunch had slightly worse glycemic control (though not significantly) compared to the others (HbA1c reduction: −1.8% before breakfast, −1.1% before lunch, −1.2% before dinner). This is likely because it is more challenging to ensure at least 6 h of fasting between breakfast and lunch if the two meals are too close together.

The improvement in the overall metabolic profile (HbA1c, BMI, lipids) observed after 3 months of therapy with oral semaglutide led to a significant reduction in cardiovascular risk, allowing a substantial percentage of patients to shift significantly from “very high” to “high” cardiovascular risk and from “high” to “moderate” risk. The PIONEER 6 trial [9] established the cardiovascular safety of oral semaglutide while the recent SOUL trial [14], with a longer follow-up of approximately 4 years, demonstrated a significant reduction in MACE (−14%), confirming the cardiovascular benefits of oral semaglutide in patients with type 2 diabetes who are at high cardiovascular risk with the Number Needed to Treat (NNT) to prevent one event of 50 persons. Our study adds novel insights by demonstrating a significant reduction in estimated cardiovascular risk, as assessed by the ESC SCORE2 and SCORE2-OP algorithms [21,22], after only 3 months of treatment with oral semaglutide, predominantly 7 mg. Although SCORE2 is a surrogate risk predictor rather than a hard clinical endpoint, our findings suggest that oral semaglutide may confer some cardiovascular benefit over a shorter duration, particularly in real-world clinical settings. Taken together, our study complements the evidence from PIONEER 6 and SOUL by suggesting that cardiovascular risk reduction with oral semaglutide may begin early in the course of treatment and may be detectable through validated risk estimation tools before long-term event data accrue. Further studies are warranted to assess whether short-term SCORE2 improvements translate into long-term cardiovascular event reduction across different dosing regimens.

Our study has limitations: first, in RWE, the choice between different therapeutic approaches is influenced by a complex evaluation of clinical, cultural, behavioral, and social aspects. Additionally, data collection may be limited by the heterogeneity in the classifications/definitions of diseases, and the collection of information may be hindered by the limited availability of data. For this reason, we did not stress or evaluate the changes in some parameters that were available only for a limited number of subjects (e.g., lipids or albuminuria).

A further limitation of the study is the lack of a control group, making it difficult to distinguish the real contribution of oral semaglutide to glycemic control and body weight from lifestyle changes (diet and physical activity) that may have occurred simultaneously. However, RCTs have clearly demonstrated the superiority of oral semaglutide over placebo in improving the glycometabolic profile. Indeed, it is unlikely that diet alone can bring about these excellent changes in weight and glycation in just three months. Third, it is also possible that by offering the patient a follow-up diabetic visit after only 3 months, the patient may have felt more motivated to improve their lifestyle to achieve the goals more quickly (trial effect?). Moreover, patients often maintain a heightened sense of accountability and are more responsive to clinical feedback, especially when they perceive immediate benefits or improvements. In addition to the shorter follow-up period of diabetic visits, the new diagnosis of diabetes may have contributed to greater improvements in glycemic control and body weight, as these conditions may have increased motivation to adopt lifestyle changes. Frequent monitoring in the short-term follow-up can serve as a form of reinforcement, providing patients with tangible evidence of their progress. Taken together, patient motivation and short-term follow-up likely could have acted synergistically in this study to optimize adherence and engagement, thereby amplifying the observed benefits of the intervention. Finally, the small sample sizes of many biochemical data may have made the statistical analysis of many parameters weaker and, therefore, these require re-evaluation.

In our study oral semaglutide showed a very good therapeutic profile, combining the efficacy of GLP-1 receptor agonists with the convenience of oral administration and a low risk of side effects, as reported in a recent review [23]. After just three months of therapy, oral semaglutide showed excellent short-term efficacy leading to a statistically significant reduction in HbA1c and body weight, and also resulting in a substantial decrease in cardiovascular risk. Given its greater efficacy in patients with a shorter duration of diabetes, this study reinforces the recommendation for the early use of GLP-1 receptor agonists.

## 4. Materials and Methods

### 4.1. Study Design

This is a multicenter, prospective, observational, single-arm study conducted between April and October 2024, involving several diabetes centers affiliated with Azienda Sanitaria 1 (ASL1) Abruzzo (L’Aquila, Avezzano, Sulmona, Castel di Sangro), Italy. Patients with type 2 diabetes mellitus and poor glycometabolic control, who received an initial prescription of oral semaglutide (T0) from April onward, were reassessed after three months (T1) to evaluate the drug’s efficacy in reducing glycated hemoglobin and body weight.

As is standard in RWE observational studies, no control group was present. The prescription of oral semaglutide was based on the principles of care and management recommended by national and international guidelines. The decision to initiate oral semaglutide, dose escalation, and administration timing (before breakfast, lunch, or dinner) was at the discretion of the prescribing physician. All patients were subjected to educational therapy (a standard diet plan for diabetes and physical activity) at diagnosis that was reinforced every time glucose control worsened, including at the time of recruitment (T0).

A series of clinical and biochemical parameters were collected both at baseline and during the follow-up diabetes consultation using electronic medical records. These parameters included demographic and anthropometric characteristics such as body weight and body mass index (BMI), diabetes duration, previous hypoglycemic therapy, cardiovascular risk class (estimated using the risk prediction model SCORE2-Diabetes [21] for patients under 70 years old and SCORE2-OP [22] for patients aged ≥ 70 years), time of oral semaglutide administration, glycated hemoglobin, creatinine, GFR (calculated using the CKD-EPI formula), lipid profile, microalbuminuria, and transaminases.

The duration of diabetes was categorized as “newly diagnosed”, “less than 5 years”, “between 5 and 10 years”, or “more than 10 years”. Cardiovascular risk was classified as “moderate”, “high”, or “very high” according to ESC (SCORE2) [21,22]; the SCORE2 was calculated by each physician at the time of the visits at T0 and T1. Individual lipid levels were not always recorded; thus, we have some missing data.

The timing of administration of oral semaglutide was recorded as “before breakfast”, “before lunch”, or “before dinner”.

The study only included patients who were receiving their first prescription of oral semaglutide and were not using other GLP-1 RAs at T0. The inclusion criteria were an age of over 18 years, a diagnosis of type 2 diabetes mellitus, a first prescription of oral semaglutide as part of any therapeutic regimen that did not include GLP-1 RAs, and the availability of glycated hemoglobin (HbA1c) and body weight data at both T0 and T1. The exclusion criteria were an age of under 18 years, other forms of diabetes mellitus, ongoing treatment with any GLP-1 RA at T0, and missing data (HbA1c and body weight). Other medications, such as lipid-lowering therapy, were not taken into account.

### 4.2. Study Endpoints

The primary endpoints of the study were the evaluation of changes in HbA1c and body weight after three months of treatment. The secondary endpoints included lipid profile improvement and cardiovascular risk category changes.

### 4.3. Statistical Analysis

Statistical analysis was performed using IBM SPSS Statistics software version 25.0, with statistical significance set at *p* < 0.05. Normal distribution of the variables was evaluated with the Shapiro–Wilk test (plots are shown in Supplementary Data). The Wilcoxon signed-rank test for non-normal distribution was applied for all variables. Categorical variables were compared using the chi-square test, and one-way ANOVA was used to compare means between groups. The Bonferroni correction for multiple comparisons was not applied because it has been shown to be problematic with non-parametric tests; for instance, it reduces power, increasing the risk of type 2 errors, especially when multiple comparisons are made. Most importantly, it assumes that multiple tests are independent, which is not true in non-parametric settings with related samples, as in our case. To assess the independent association of selected variables at baseline (age, BMI, duration of diabetes, creatinine, and triglycerides) with changes in HbA1c at follow-up, a linear multivariate analysis was performed.

Continuous variables are reported as the mean (±standard deviation) or as the median (and interquartile range), while categorical variables are reported as a number and percentage.

## 5. Conclusions

After just three months of therapy, oral semaglutide showed excellent short-term efficacy, leading to a significant reduction in HbA1c and body weight, and also resulting in a substantial decrease in cardiovascular risk. It showed greater efficacy in patients with a shorter duration of diabetes, reinforcing the recommendation of the early use of GLP-1 receptor agonists in the treatment of type 2 diabetes.

## Figures and Tables

**Table 1:** Characteristics of population at baseline (T0) and three months after start of oral semaglutide (T1). *p*-value refers to difference between T0 and T1. Data are expressed as median (interquartile range) when not normally distributed (Shapiro–Wilk test).

	Available (%)	T0	T1	*p* Value
Age (years)	100%	69 (12)		
Gender	100%	Males: 90 (54%) Females: 77 (46%)		
Duration of diabetes	100%	7.6 ± 4.2		
Body weight (kg)	100%	83.3 (19)	80 (20.5)	0.000
BMI (kg/m^2^)	100%	29.6 (7.1)	28.6 (6.8)	0.000
HbA1c (%)	100%	8.1 (2)	6.9 (1.1)	0.000
Glycemia (mg/dL)	50.3%	161 (68)	123 (30)	0.134
Creatinine (mg/dL)	61.1%	0.85 (0.31)	0.92 (0.18)	0.147
Total cholesterol (mg/dL)	34.7%	169 (54)	139 (37.5)	0.000
HDL cholesterol (mg/dL)	37.1%	46 (16.5)	43 (16)	0.000
Triglycerides (mg/dL)	35.9%	127 (83.5)	119 (81)	0.060
LDL cholesterol (mg/dL)	35.3%	91 (40.5)	71 (32.8)	0.000
Albuminuria (mg/g)	13.8%	11 (28)	5 (26)	0.039
GOT (U/L)	17.3%	24 (18)	21 (9)	0.008
GPT (U/L)	17.9%	27.5 (19)	21 (15)	0.002

BMI, body mass index; HDL, high-density lipoprotein; LDL, low-density lipoprotein; GOT, glutamic oxaloacetic transaminase; GPT, glutamic pyruvic transaminase.

**Table 2:** Change in mean HbA1c (Δ) between T1 and T0 in different patient groups.

		Total 167	Change in Mean HbA1c (Δ)	*p* Value
Percentage of body weight loss	<5%	116 (69.5%)	1.1 ± 1.4	0.012
	≥5%	51 (30.5%)	1.9 ± 1.7	
Timing of drug administration	Before breakfast	29 (17.4%)	1.8 ± 1.8	0.296
	Before lunch	60 (35.9%)	1.1 ± 1.6	
	Before dinner	78 (46.7%)	1.2 ± 1.3	

**Table 3:** Multivariate linear regression analysis. Dependent variable is HbA1c T1.

Variable	Unstandardized Coefficients		Standardized Coefficients		
	β	Std. Error	β	t	*p*-Value
(Constant)	5.524	1.517		3.641	0.001
HbA1c T0 (%)	0.189	0.085	0.271	2.228	0.029
BMI (kg/m^2^)	−0.003	0.025	−0.014	−0.11	0.913
Creatinine (mg/dL)	−0.394	0.498	−0.106	−0.791	0.431
Age (years)	0.005	0.011	0.05	0.404	0.688
Gender	−0.165	0.289	−0.081	−0.571	0.57
DM < 5 years	0.268	0.119	0.261	2.248	0.028
Triglycerides (mg/dL)	−0.002	0.002	−0.153	−1.236	0.221

## Data Availability

The data presented in this study are available from the corresponding author upon request. The data are not publicly available due to privacy restrictions and the lack of specific patient consent.

## Supplementary Materials

The following supporting information can be downloaded at: https://www.mdpi.com/article/10.3390/ph18060856/s1, Supplementary data: Shapiro-Wilk test for normality.

## Abbreviations

The following abbreviations are used in this manuscript:

GLP1-RA	GLP1-receptor agonist
HbA1c	Glycated hemoglobin
T2DM	Type 2 diabetes mellitus
MACE	Major adverse cardiovascular events
RCTs	Randomized controlled trials
RWD	Real-world data
RWE	Real-world evidence
CV	Cardiovascular
BMI	Body mass index
GOT	Glutamic oxaloacetic transaminase
GPT	Glutamic pyruvic transaminase
GFR	Glomerular filtration rate
NNT	Number Needed to Treat
