# Supplementary material for: Real-World Analysis of Short-Term Effectiveness of Oral Semaglutide: Impact on Glycometabolic Control and Cardiovascular Risk
Source: Pharmaceuticals (Basel). 2025 Jun 8;18(6):856. doi: 10.3390/ph18060856 (PMC12195834; doi:10.3390/ph18060856)

Shapiro-Wilk test for normality

AGE (years)

	Kolmogorov-Smirnov[a]			Shapiro-Wilk		
	Statistica	gl	Sign.	Statistica	gl	Sign.
Età Precisa	,121	121	,000	,950	121	,000

a. Correzione di significatività di Lilliefors

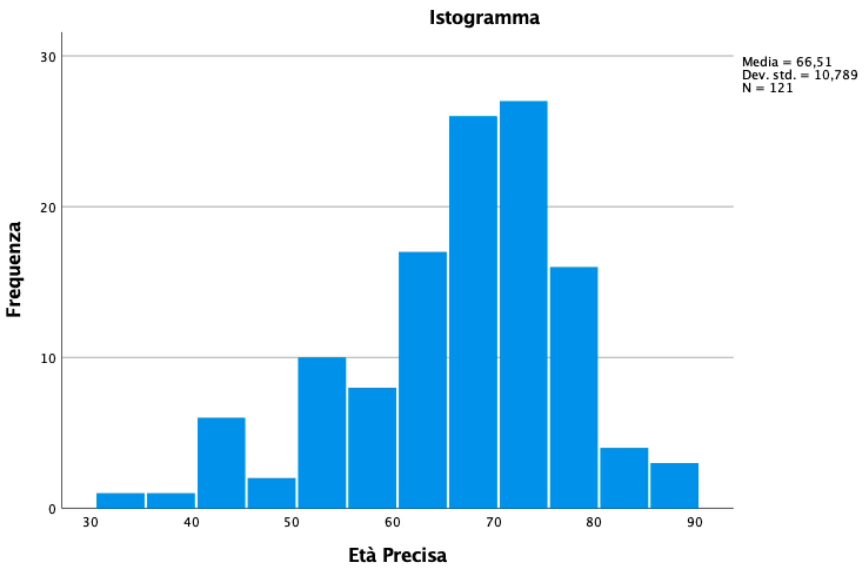

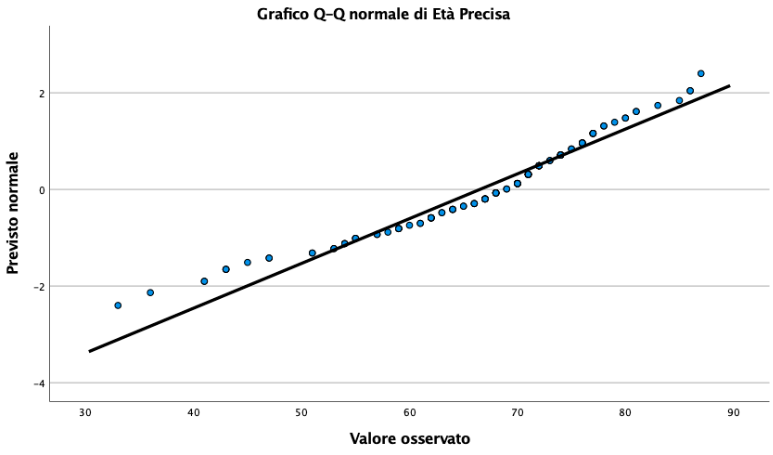

Body weight (kg)

	Kolmogorov-Smirnov[a]			Shapiro-Wilk		
	Statistica	gl	Sign.	Statistica	gl	Sign.
PESO T0	,078	167	,015	,949	167	,000

a. Correzione di significatività di Lilliefors

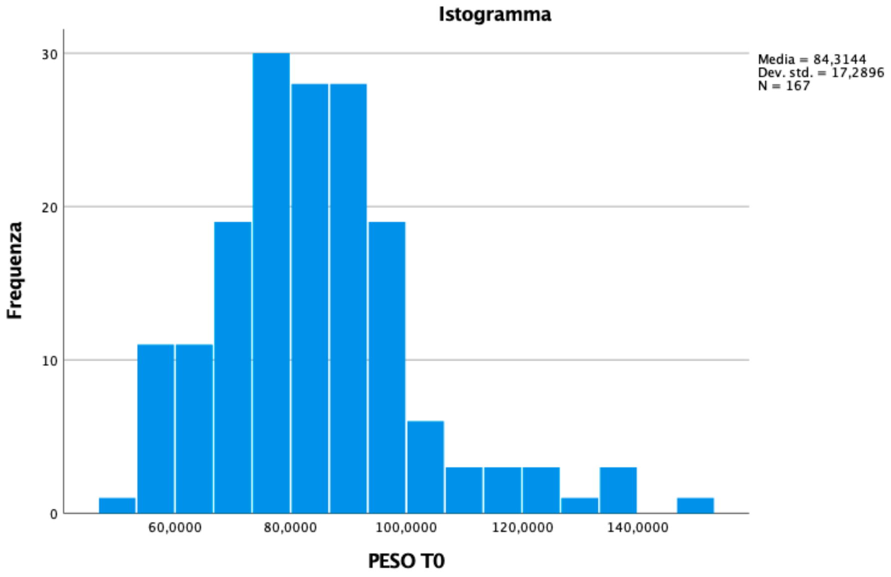

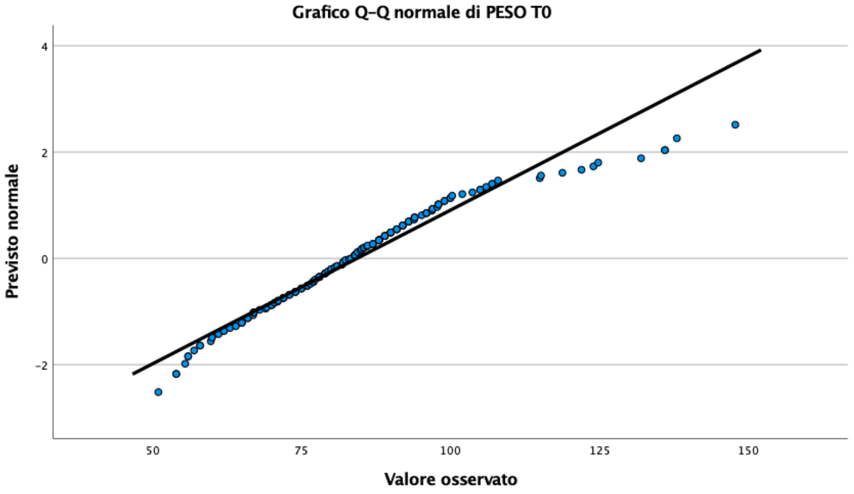

<u>BMI (kg/m2)</u>

	Kolmogorov-Smirnov[a]			Shapiro-Wilk		
	Statistica	gl	Sign.	Statistica	gl	Sign.
BMI T0	,100	167	,000	,949	167	,000

a. Correzione di significatività di Lilliefors

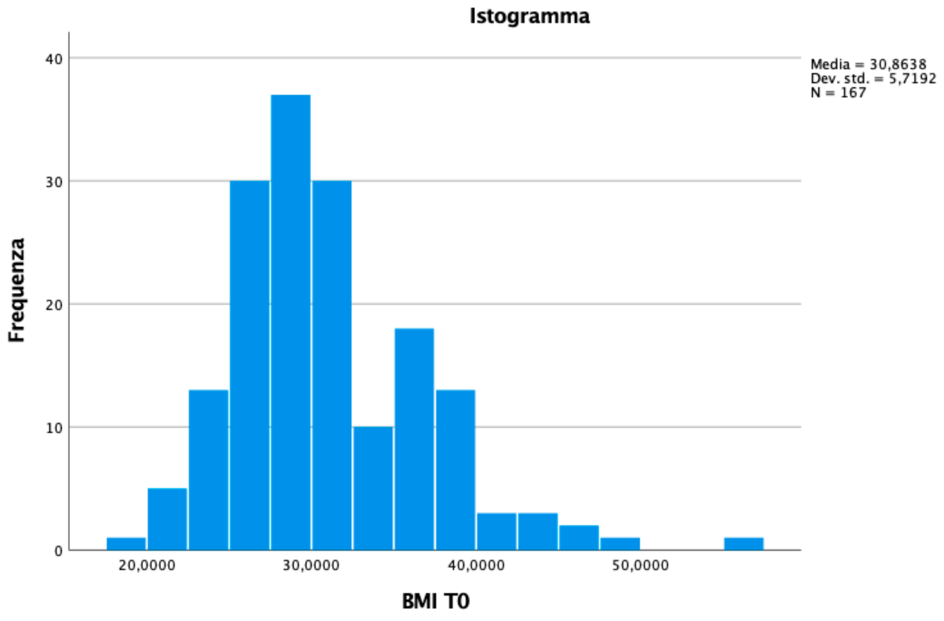

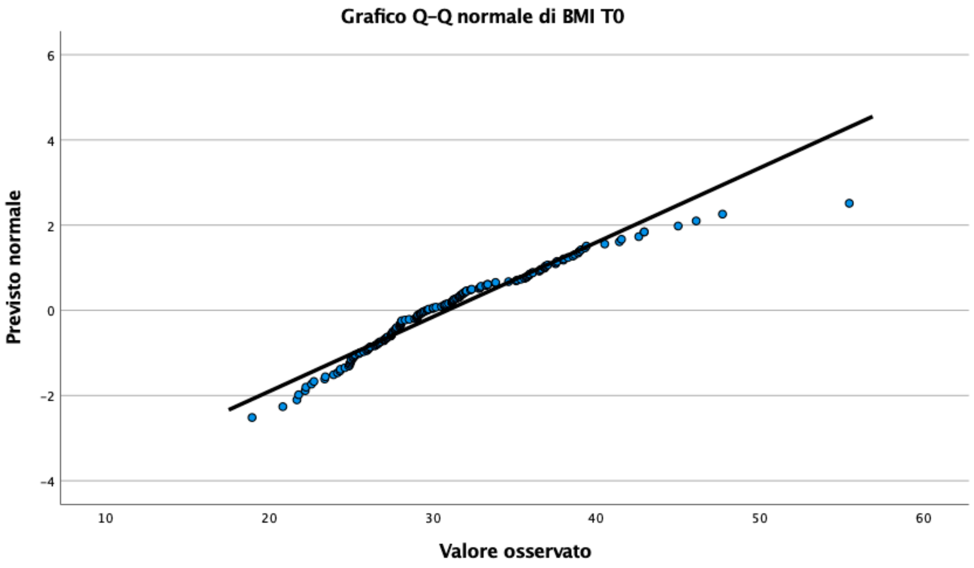

HbA1c (%)

	Kolmogorov-Smirnov[a]			Shapiro-Wilk		
	Statistica	gl	Sign.	Statistica	gl	Sign.
COMPENSO METABOLICO T0 (GLICATA)	,112	167	,000	,944	167	,000

a. Correzione di significatività di Lilliefors

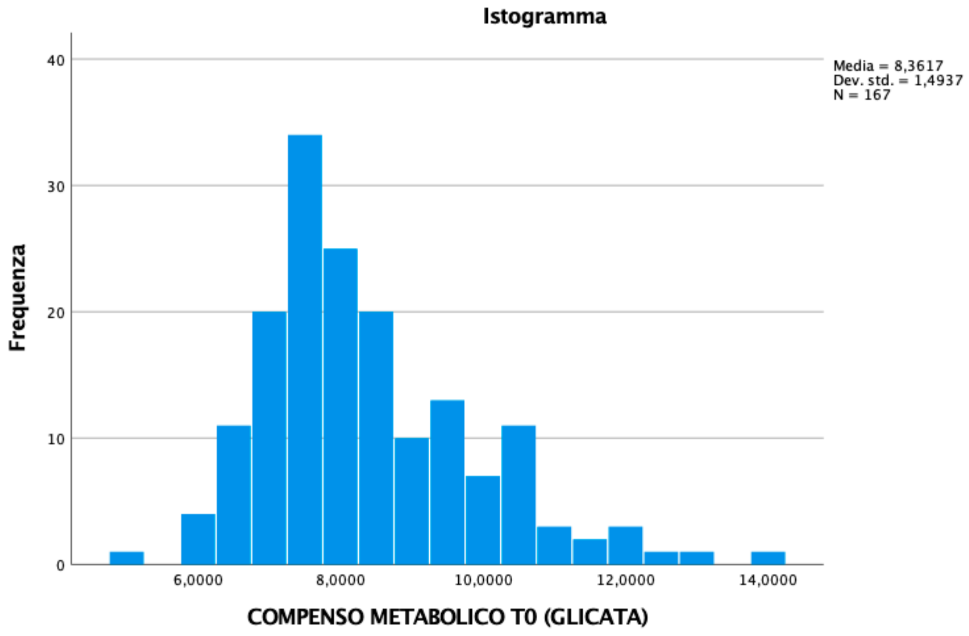

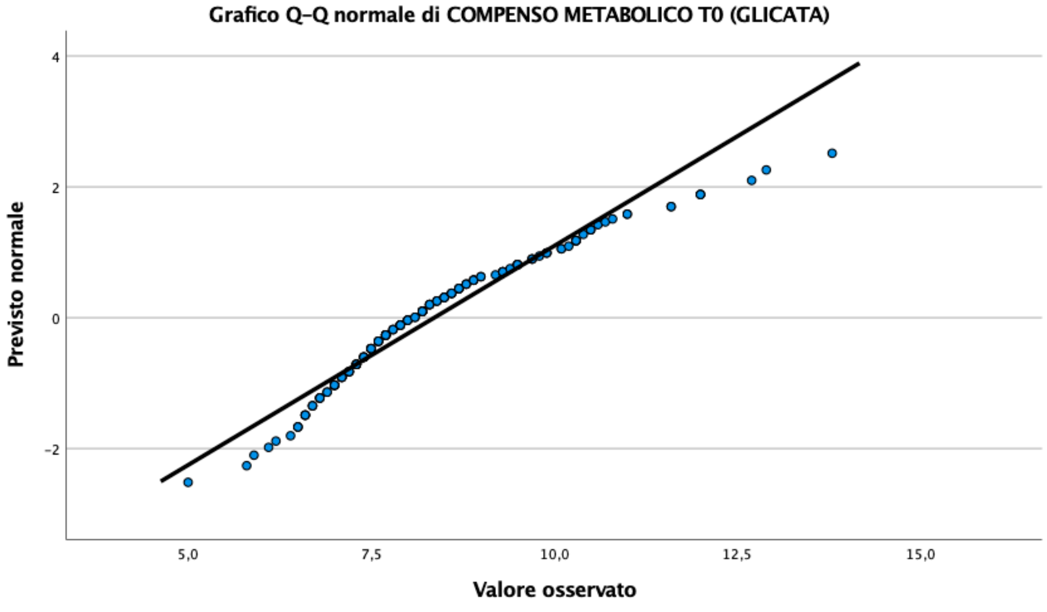

Glycemia (mg/dl)

	Kolmogorov-Smirnov[a]			Shapiro-Wilk		
	Statistica	gl	Sign.	Statistica	gl	Sign.
Glicemia a digiuno T0	,115	97	,003	,913	97	,000

a. Correzione di significatività di Lilliefors

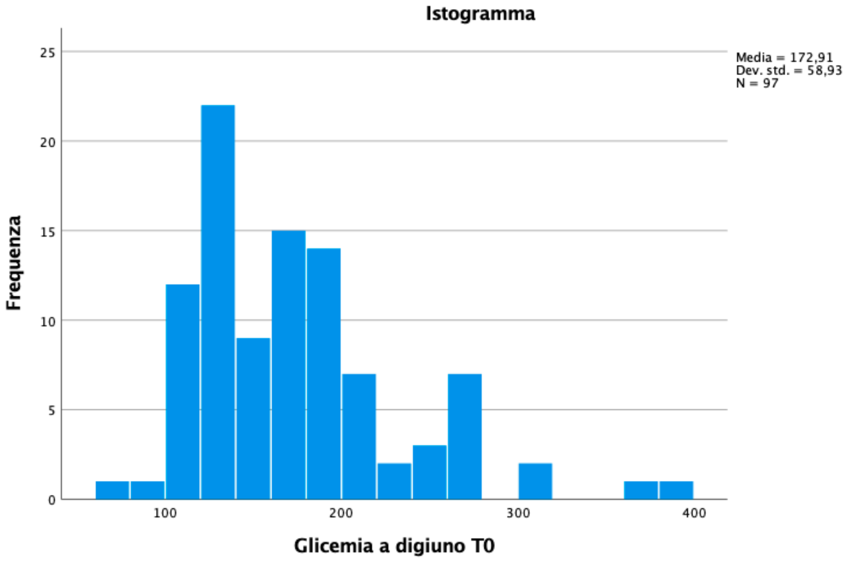

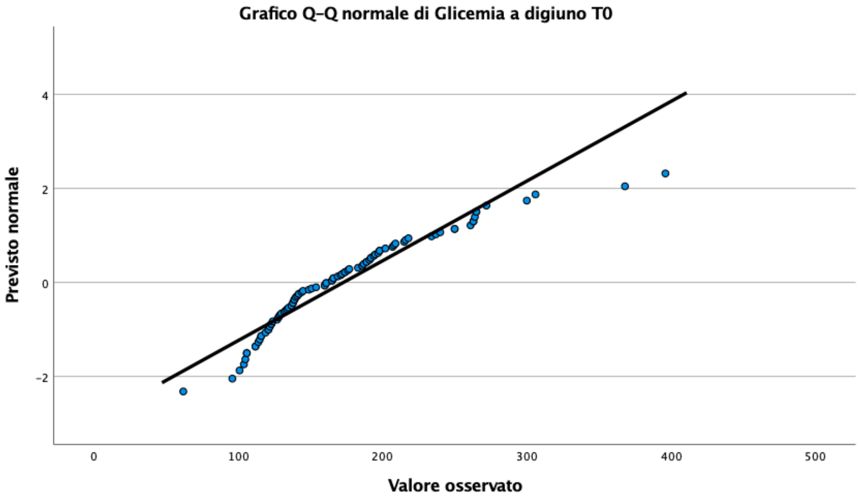

Creatinine (mg/dl)

	Kolmogorov-Smirnov[a]			Shapiro-Wilk		
	Statistica	gl	Sign.	Statistica	gl	Sign.
Creatinina T0	,120	106	,001	,876	106	,000

a. Correzione di significatività di Lilliefors

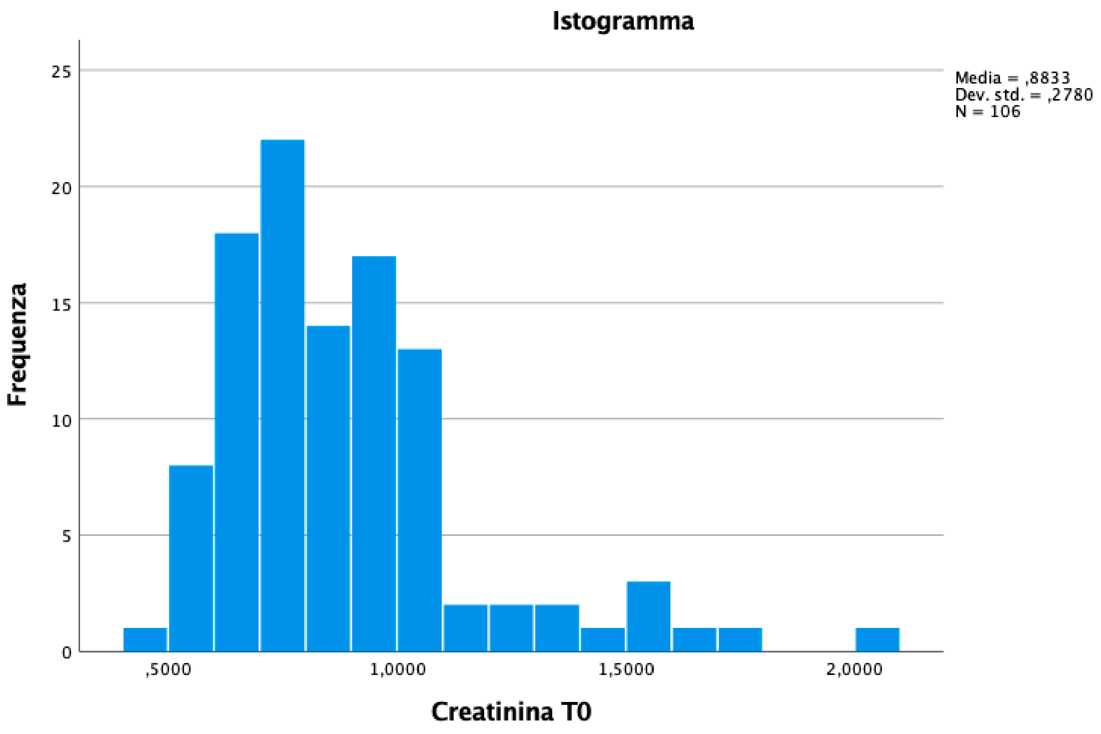

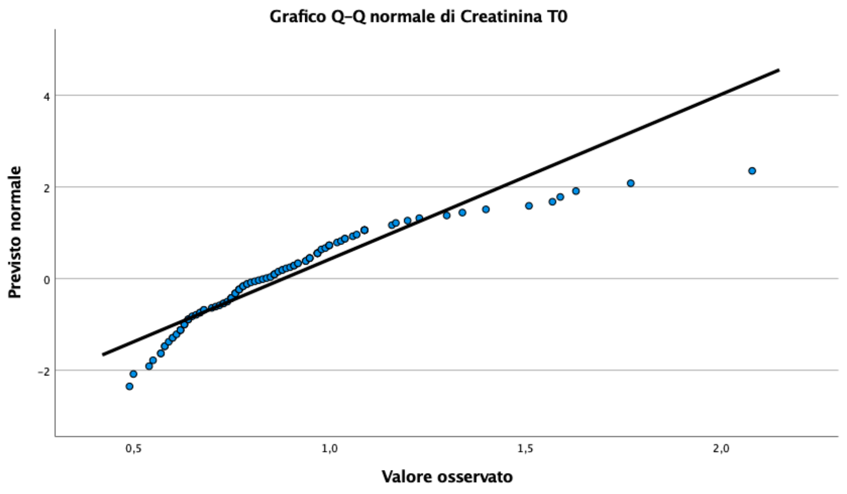

Total cholesterol (mg/dl)

	Kolmogorov-Smirnov[a]			Shapiro-Wilk		
	Statistica	gl	Sign.	Statistica	gl	Sign.
Colesterolo totale T0	,103	80	,036	,955	80	,007

a. Correzione di significatività di Lilliefors

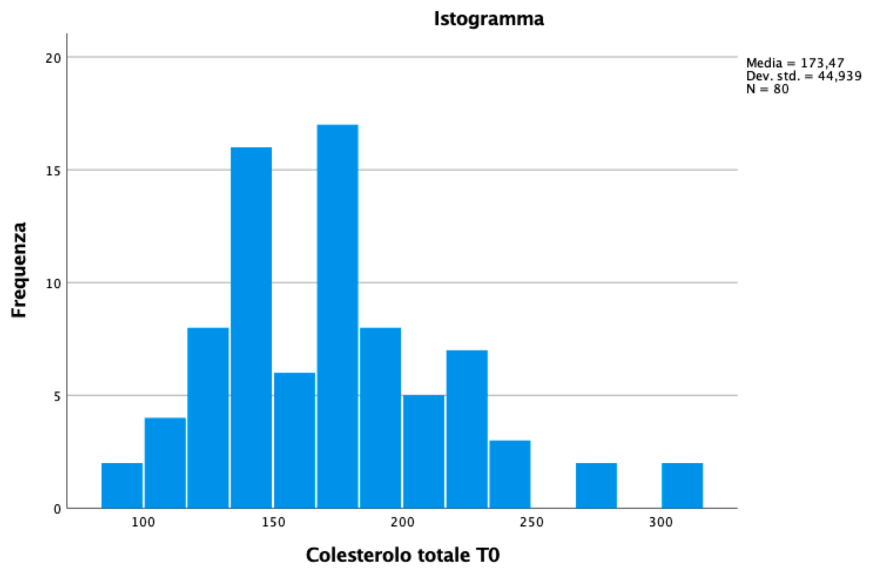

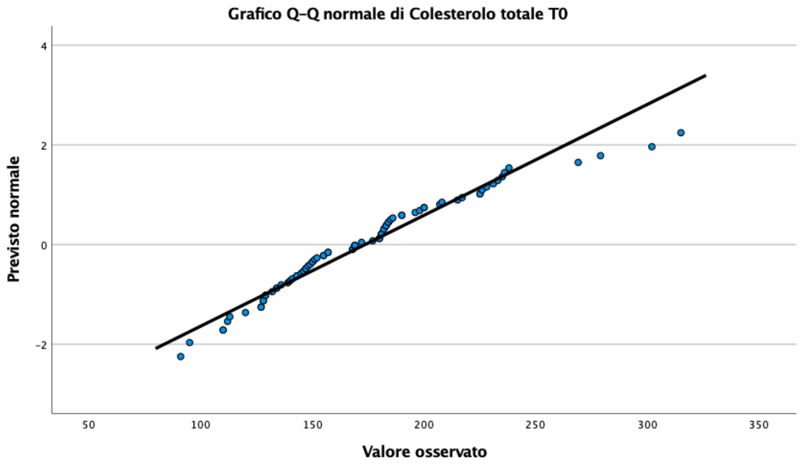

HDL cholesterol (mg/dl)

	Kolmogorov-Smirnov[a]			Shapiro-Wilk		
	Statistica	gl	Sign.	Statistica	gl	Sign.
HDL T0	,108	79	,024	,941	79	,001

a. Correzione di significatività di Lilliefors

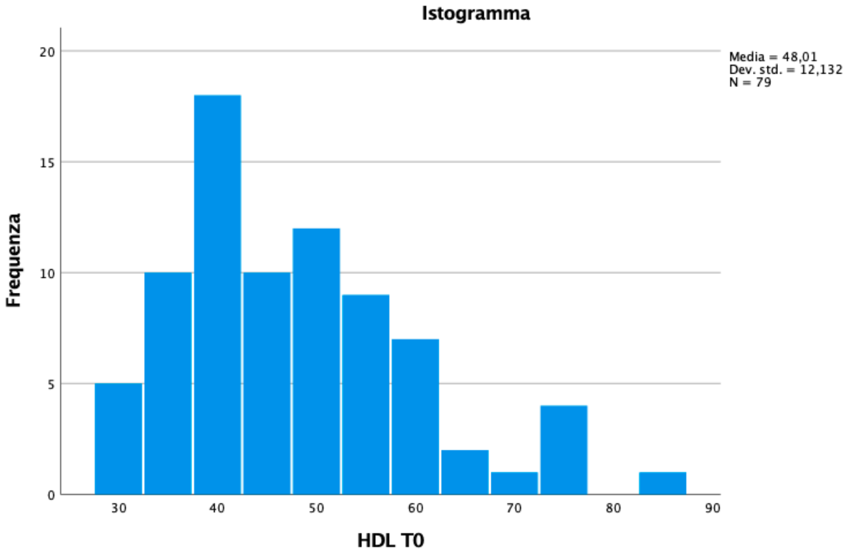

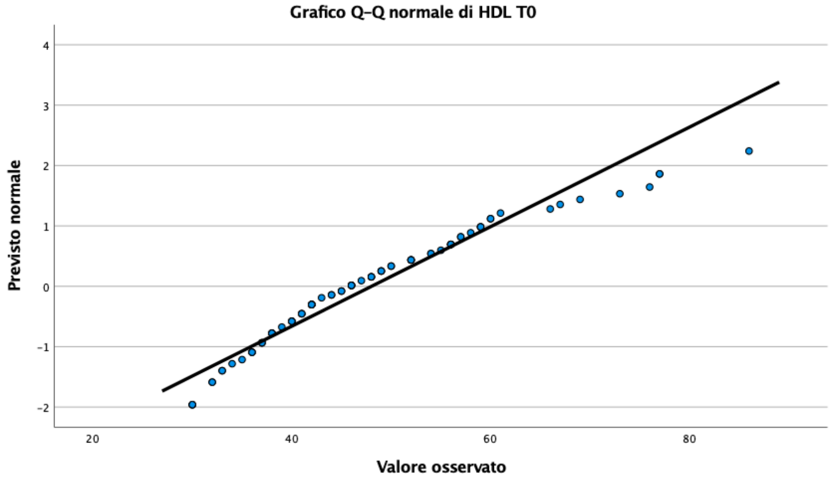

Triglycerides (mg/dl)

	Kolmogorov-Smirnov[a]			Shapiro-Wilk		
	Statistica	gl	Sign.	Statistica	gl	Sign.
Trigliceridi T0	,131	83	,001	,874	83	,000

a. Correzione di significatività di Lilliefors

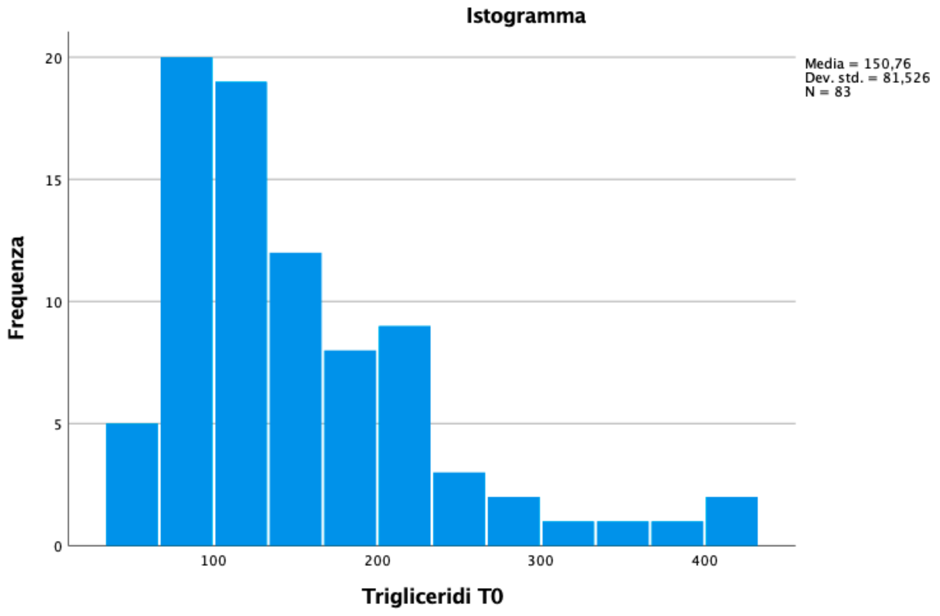

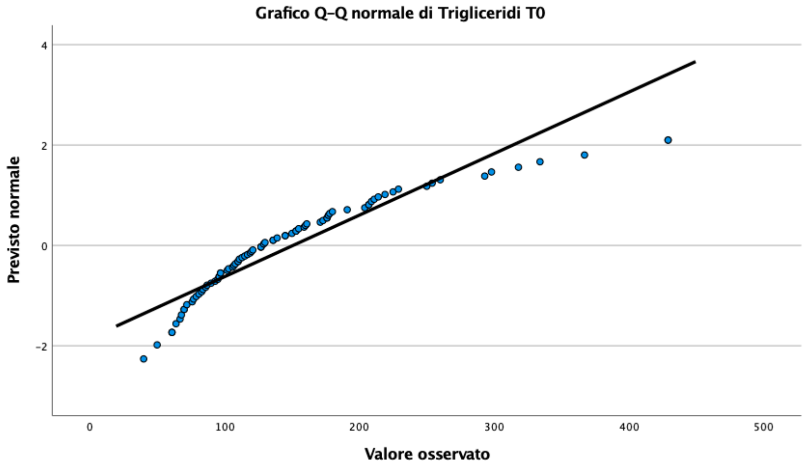

LDL cholesterol (mg/dl)

	Kolmogorov-Smirnov[a]			Shapiro-Wilk		
	Statistica	gl	Sign.	Statistica	gl	Sign.
LDLc T0	,076	82	,200*	,954	82	,005

*. Questo è un limite inferiore della significatività effettiva.

a. Correzione di significatività di Lilliefors

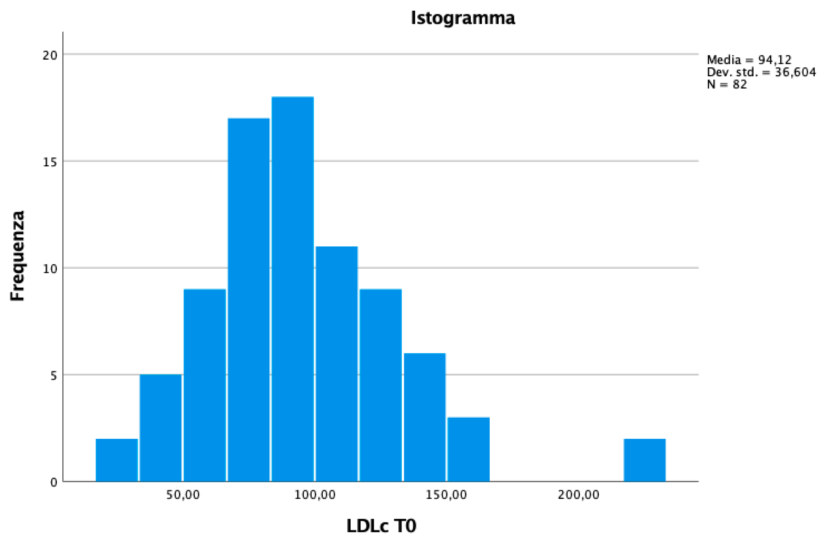

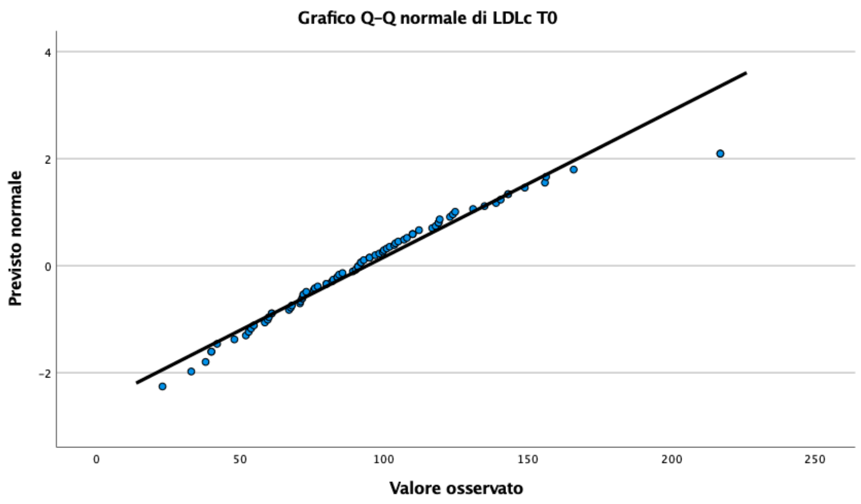

Albuminuria (mg/g)

	Kolmogorov-Smirnov[a]			Shapiro-Wilk		
	Statistica	gl	Sign.	Statistica	gl	Sign.
Microalbuminuria T0	,409	51	,000	,299	51	,000

a. Correzione di significatività di Lilliefors

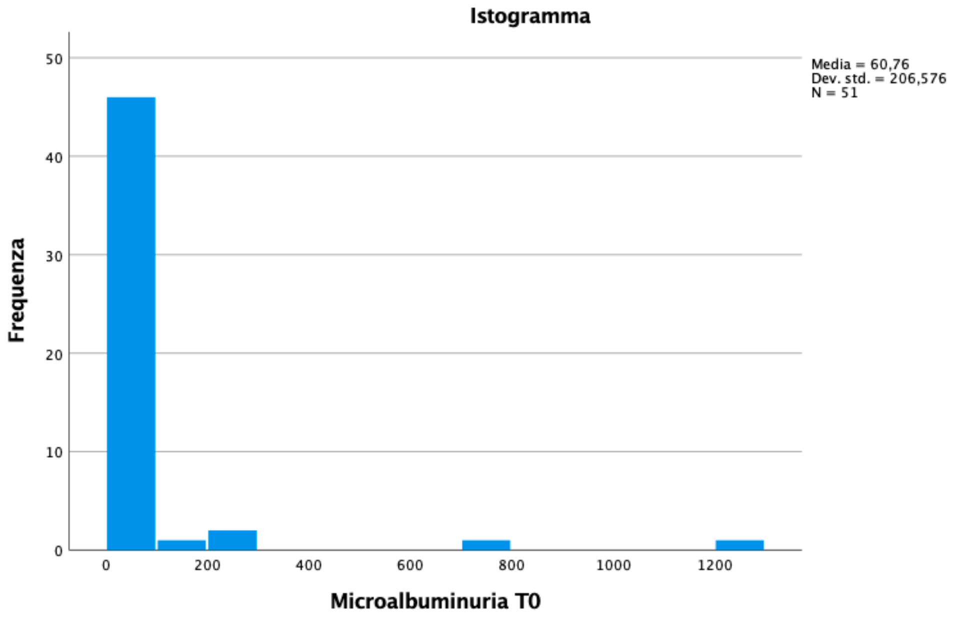

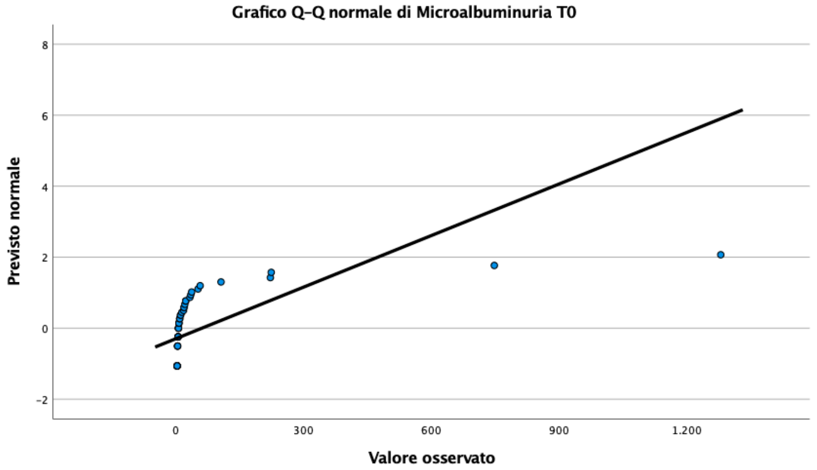

GOT (U/l)

	Kolmogorov-Smirnov[a]			Shapiro-Wilk		
	Statistica	gl	Sign.	Statistica	gl	Sign.
GOT T0	,186	54	,000	,818	54	,000

a. Correzione di significatività di Lilliefors

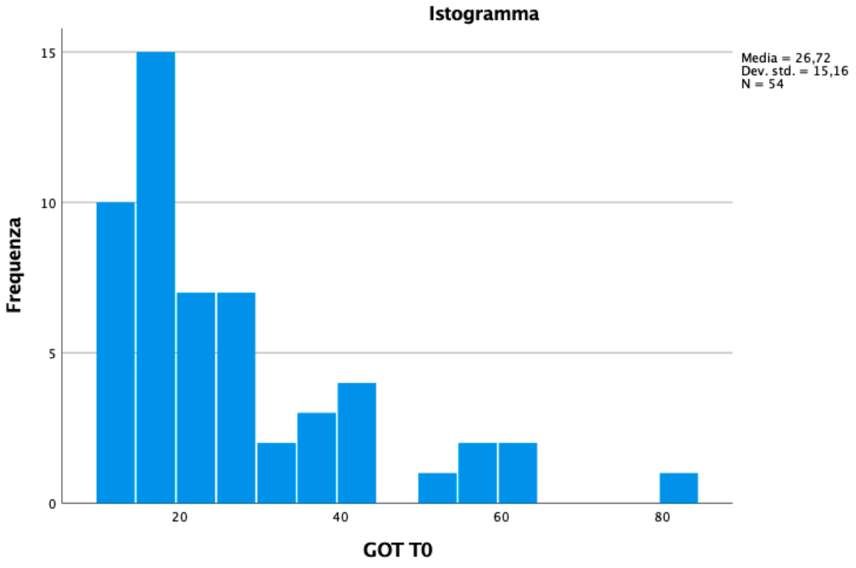

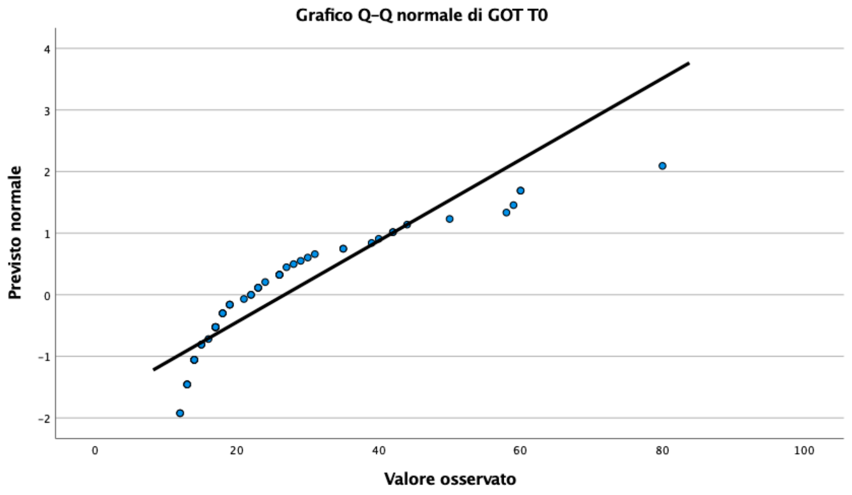

GPT (U/l)

Test di normalità

	Kolmogorov-Smirnov[a]			Shapiro-Wilk		
	Statistica	gl	Sign.	Statistica	gl	Sign.
GPT T0	,156	57	,001	,857	57	,000

a. Correzione di significatività di Lilliefors

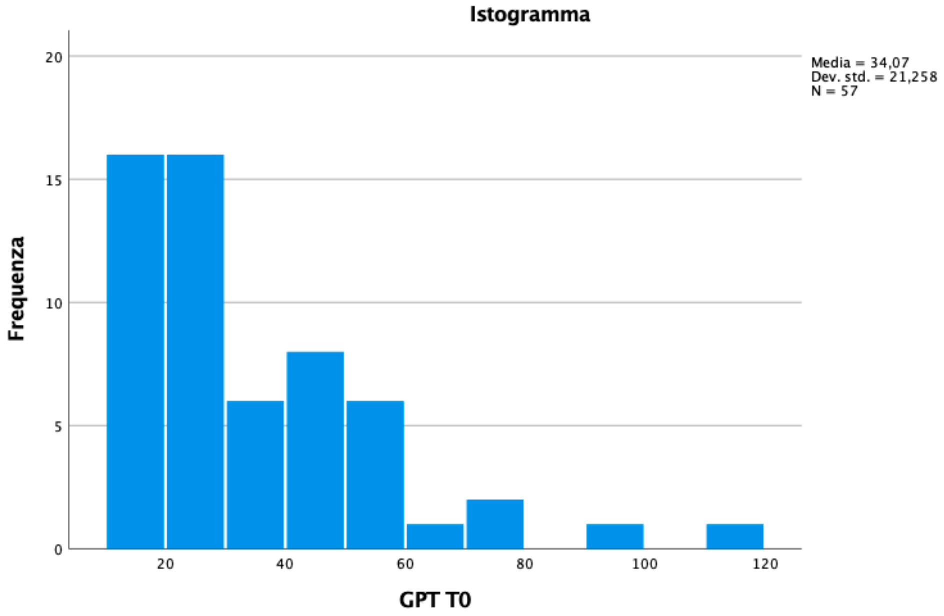

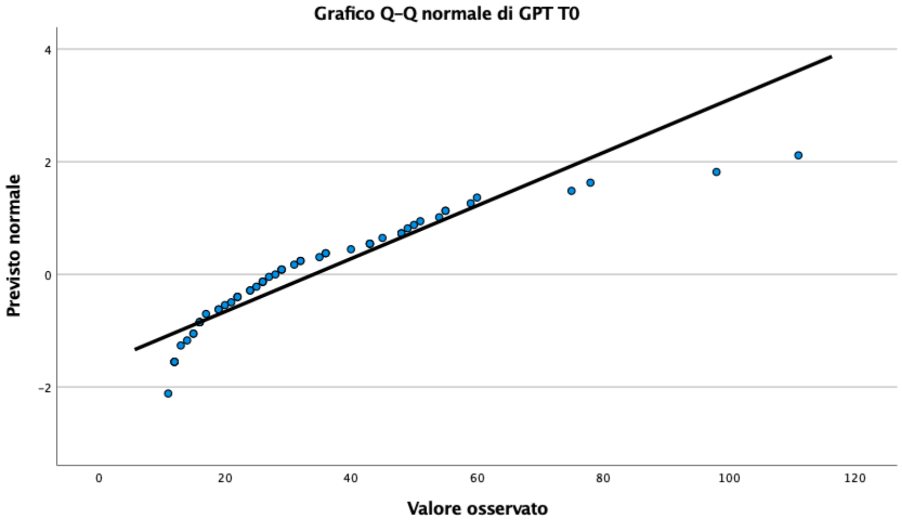

Supplement: Supplementary file 1 [file pharmaceuticals-18-00856-s001.zip › pharmaceuticals-3635038-supplementary.pdf]
